# Morbidity profile and sociodemographic characteristics of unaccompanied refugee minors seen by paediatric practices between October 2014 and February 2016 in Bavaria, Germany

**DOI:** 10.1186/s12889-018-5878-7

**Published:** 2018-08-07

**Authors:** Teresa Kloning, Thomas Nowotny, Martin Alberer, Michael Hoelscher, Axel Hoffmann, Guenter Froeschl

**Affiliations:** 1 Swiss Tropical and Public Health Institute, Basel, Switzerland; 2 University of Basel, Basel, Switzerland; 3 Privatärztliche Kinder- und Jugendarztpraxis, Stephanskirchen, Germany; 4 Division of Infectious Diseases and Tropical Medicine, Medical Centre of the University of Munich (LMU), Munich, Germany

**Keywords:** Unaccompanied refugee minors, Morbidity, Germany, Immunity, Sociodemographics

## Abstract

**Background:**

This study aimed to investigate the morbidity profile and the sociodemographic characteristics of unaccompanied refugee minors (URM) arriving in the region of Bavaria, Germany, between October 2014 and February 2016.

**Methods:**

The retrospective cross sectional study included 154 unaccompanied refugee minors between 10 and 18 years of age. The data was derived from medical data records of their routine first medical examination in two paediatric practices and one collective housing for refugees in the region of Bavaria, Germany.

**Results:**

Only 12.3% of all participants had no clinical finding at arrival. Main health findings were skin diseases (31.8%) and mental disorders (25%). In this cohort the hepatitis A immunity was 92.8%, but only 34.5% showed a constellation of immunity against hepatitis B. Suspect cases for tuberculosis were found in 5.8% of the URM. There were no HIV positive individuals in the cohort. Notably, 2 females were found to have undergone genital mutilations.

**Conclusions:**

The majority of arriving URM appear to have immediate health care needs, whereas the pathologies involved are mostly common entities that are generally known to the primary health care system in Germany. Outbreaks due to hepatitis A virus are unlikely since herd immunity can be assumed, while this population would benefit from hepatitis B vaccination due to low immunity and high risk of infection in crowded housing conditions. One key finding is the absence of common algorithms and guidelines in health care provision to URM.

## Background

In the year 2015 there were 21.3 million people forced to leave their home countries worldwide. The majority of the refugees (54%) came from the following three countries: the Syrian Arab Republic with 4.9 million, Afghanistan with 2.7 million and Somalia with 1.1 million [1].

In Germany the numbers of asylum seekers increased substantially in recent years, culminating in a peak influx of refugees into the German state of Bavaria towards the end of 2015. According to Eurostat the number of asylum applications by unaccompanied refugee minors (URM) in Germany more than quintupled (from 4400 to 22.255) from 2014 to 2015 [2]. More detailed data on the distribution of URM across different German regions are not available [3], which is also due to the unpreparedness of both the German authorities and the German health infrastructure prevalent at peak times in 2015. However, investigative journalism published an article about the dramatic rise of arriving URM in Bavaria. While in 2013 just above 500 URM arrived to Bavaria, in 2014 there were already 3400 and finally in 2015 16,800 arrivals [4]. This development also challenged the public health care system.

According to an assessment by the European Centre for Disease Prevention and Control the refugees are considered to be generally in a good health status at the beginning of their journey [5]. However, different conditions can have an impact on their health status at arrival, like for example traumas from war, or having experienced violence and torture in the war zone [6], as well as disrupted health care in their home countries [7]. Additional determinants of ill health follow in the course of the trajectory, such as adverse circumstances of the travel like being isolated, unsupported, or being forced to undergo physical strain or dangers. Poor living conditions, poor nutrition, inadequate hygienic conditions, overcrowded refugee camps and lack of health care services are other relevant factors for unfavourable health outcomes [8]. Within the refugee population URM are a particularly vulnerable group, because they are lacking protection or support from their families or relatives [9].

Several recent studies from Germany and other countries receiving refugees in Europe have covered the prevalence of communicable and non-communicable diseases in arriving refugees, mostly in adult populations. Just a few studies on refugee minors have been published so far. Mockenhaupt et al. have found a prevalence of intestinal parasites of 22% in 488 Syrian URM in Berlin, whereas 66% were considered to be healthy in this cohort [10]. In a study by Spallek et al. on 102 URM in Bielefeld, 58.8% of the participants were carriers of at least one infectious disease. Furthermore in 7.9% a chronic hepatitis B infection was diagnosed [11]. After arriving in the host country the risk of transmission of communicable diseases within the refugee population is high, particularly in case of late diagnosis and therapy. One of the key reasons is the often inadequate and overcrowded living conditions, as it has been described e.g. for a measles outbreak in a refugee camp in France [12]. Therefore the early initial medical examination for URM is crucial in order to be able to address preventable infections for example by administration of vaccines, and to identify existing diseases and to treat them accordingly. International law, like the convention on the right of children, requires that all actions concerning children should be in the best interests of the children themselves [13], whereby the medical examination can be considered as a key element for the protection of child health.

Recipient countries like Canada or Australia developed already years ago very detailed guidelines to support primary health care practitioners in an adequate handling with medical examinations of new arriving refugees [14–16]. For the given moment similar tools did not exist for Germany. A systematic literature research by Hvass et al.on the health screening of refugees showed host countries, like North America, Australia/New Zealand and Europe, concentrate typically on infectious diseases rather than on mental health or non-infectious diseases [17].

For the current refugee health situation in Germany there is a growing wealth of data on disease prevalence in arriving refugee populations in general, however, it has to be mentioned that data on the morbidity profile in URM is scarce [18]. In order to contribute to the availability of data on prevalence of medical conditions and immune status of URM this study has been designed and conducted.

## Methods

### Study design

This is a retrospective cross-sectional study. Anonymized medical data from paper based files of newly arrived URM were analyzed. The data from the initial medical examination after arriving to Germany was collected in two specialized practices for paediatrics (location A and location B) and in a collective housing for refugees (a former hotel complex, location C) in the state of Bavaria (South Germany). All examining physicians were specialized paediatricians.

Data was collected from October 2014 until February 2016. The initial medical examination was done regardless of existing signs of illness as stipulated by Section 62 of the German Asylum Act. Hence the examinations were mandatory. All examinations were conducted under language barriers, since URM were generally not speaking any German. However interpreters were provided occasionally by the Youth Welfare Office, while in other cases fellow refugees were translating into English. The Youth Welfare Office was at the same time acting as the legal representative conveying consent to the medical interventions conducted.

All three performing parties used their own self developed examination form to record the information. Therefore in each sub-cohort the range of collected variables is different.

Information on sex, age, country of origin and the escape route (via sea or land) were collected. Since age was recorded in full years only, some URM that were 17 years of age but had their birthday that same year are classified as 18 years of age. All screening contacts were conducted under substantial time constraints by the executing physicians.

### Data collection tools

All collected data were entered at the different sites onto paper-based data collection forms. A physical examination was performed. Blood samples were tested for hepatitis A, B and C, human immunodeficiency virus (HIV) and syphilis (Treponema pallidum haem-agglutination test; TPHA). In addition, a differential blood count was done. Stool diagnostics were performed and included an antigen test through an enzyme immunoassay for Helicobacter pylori (not performed in location C), amoeba, lamblia and microscopy for parasites.

The URM underwent diagnostic tests for tuberculosis (Tb), either through an Interferone Gamma Release Assay (Quantiferon Gold Test) which was envisaged for URM below 16 years (only performed in location A and B). In URM of 16 years and above a chest X-Ray was the standard screening measure. However, chest X-Ray results are only available for location B, since adequate feedback mechanisms were missing in locations A and C. Testing for Tb was not performed in location C. In suspect cases a referral to a pulmonologist was ordered. In location A previous contact to known Tb patients was documented.

### Definitions of variables

In location A and B the resultsfor Eosinophilia were documented only as pathological or normal. The threshold for a pathological finding for Eosinophils was 4%. The range of normal for leucocyte counts was 4.0–10.0 × 10^3^/μl and for thrombocyte counts 130–350 × 10^3^/μl. The results for mean corpuscular volume (MCV) were coded as pathological or normal in all three locations. Erythrocytic microcytaemia was defined as MCV below defined threshold levels. The applied thresholds were different across locations. Location A and B had a lower limit of 76.7 fl, whereas in location C the threshold for URM below 17 years was 79 fl, and for 17 years and above 80 fl.

The thresholds for haemoglobin (Hb) were as well different across locations. In location A and B the results for Hb were documented only as pathological or normal. Location A and B had a lower limit of 11.0 g/dl, whereas in location C the threshold for URM below 16 years was 12.8 g/dl, and for 16 years and above 13.5 g/dl.

The divergent thresholds for MCV and Hb had to be sustained in the course of the analysis, as data was only available in a dichotomous format as above or below threshold (except for Hb in location C, where precise results are available).

### Psycho-social questionnaire

A psycho-social questionnaire was applied to the URM at location A. This tool has been designed by the responsible physician based on personal experiences in psychosocial assessment of paediatric cases. The physician expressed a suspicion of post traumatic stress disorder (PTSD) based on his overall clinical judgement and presence of three key components of the questionnaire: sleeping disorders, nightmares and frequent headaches. In location B the responsible physician has also expressed suspicions of PTSD, however, the assessment algorithm was not found to be reproducible, the cases from location B are therefore not included. It has to be pointed out that the data on findings in location A are indicating an overall magnitude of distress in the target population as a whole, potentially needing structured intervention at later stages, rather than individual diagnoses.

### Data analysis

For the data analysis the data of all three locations were combined and analysed as a cohort at large as the different sites examined comparable target populations in the same geographical area.

Data was entered into an Excel 2010 database. Data entry was double checked by cross-checking of paper files and the digital database. The database was imported and analysed by using the statistical software Stata SE14.

### Ethical considerations

The study was approved by the Health Department of the District Office of Rosenheim, where the study has taken place.The District Office has been acting as legal guardian of the involved unaccompanied refugee minors. In addition, the study has been granted ethical clearance by the Ethics Committee of the Ludwig-Maximilians-Universität, Munich (opinion number 462–16).

## Results

**Fig. 1:**
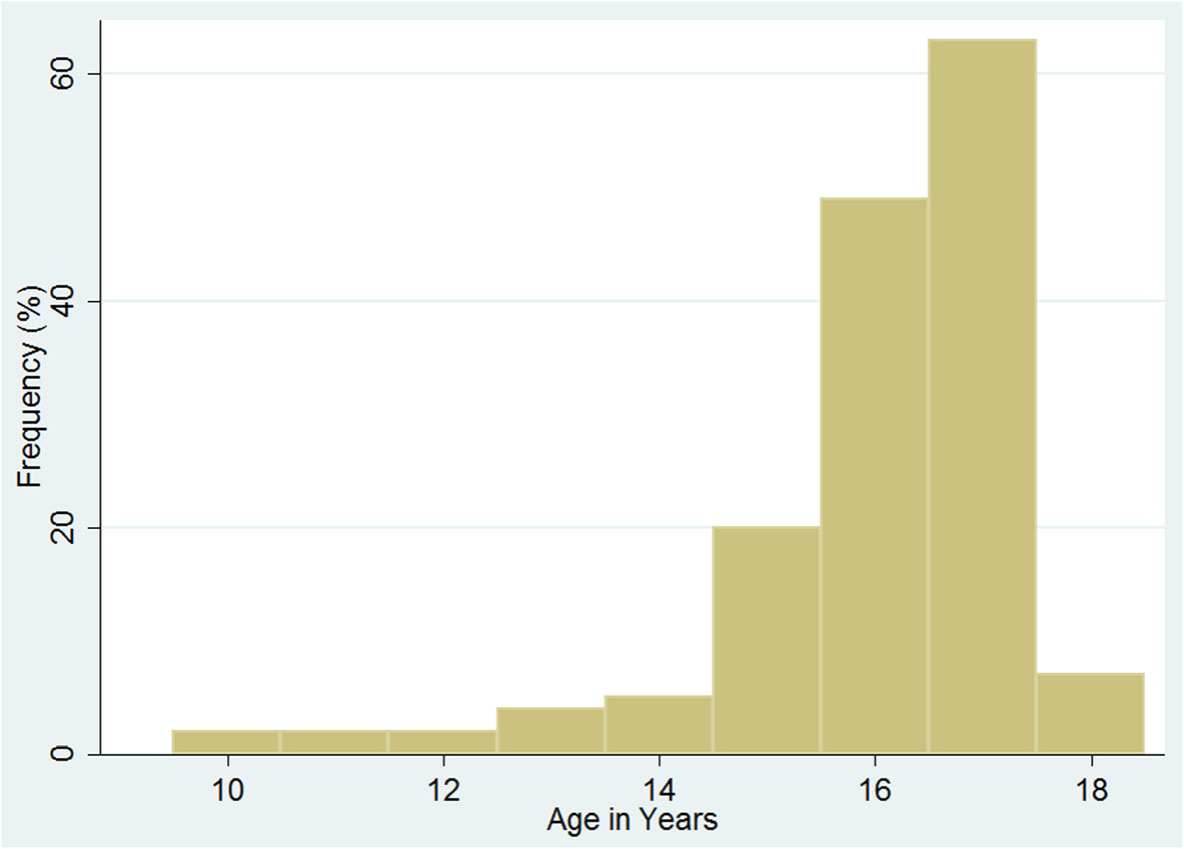
Age distribution. The histogram is representing the recorded age distribution in the entire study population

This study included 154 URM, of which 5.8% (*n* = 9/154) were female and 93.5% (*n* = 144/154) were male participants; for one person the sex was not recorded (0.7%, 1/154). The recorded age range was from 10 to 18 years at the moment of the first medical check-up, with a median age of 16 years (males: median age = 16, range 10 to 18; female: median age = 17, range 14 to 18). Figure 1 is representing the recorded age distribution of the entire study population.

**Fig. 2:**
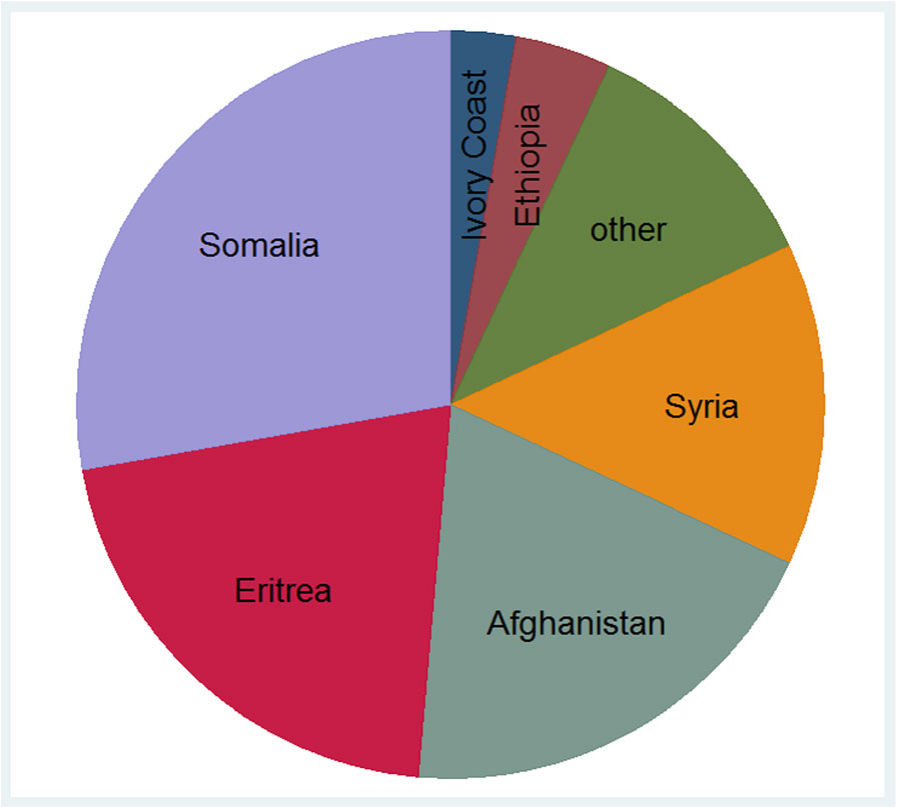
Countries of origin. The category “other” combines all countries of origin that were stated only once. These countries are: Algeria, Gambia, Ghana, Guinea, Mali, Nigeria, Pakistan and Togo

The country of origin was documented for 46.7% (*n* = 72/154) of the children from locations A and B, whereas in location C the country of origin was not recorded. The URM came from 14 different countries. The main countries were Somalia with 27.8% (*n* = 20/72), Eritrea 20.8% (*n* = 15/72) and Afghanistan 19.4% (*n* = 14/72), followed by Syria 13.9% (*n* = 10/72) (Fig. 2). As far as the data is available the female children came only from two countries: Somalia (*n* = 4/9) and Eritrea (*n* = 4/9).

For 48 URM (31.2%) the routes of passage were documented. 83.3% (*n* = 40/48) of these URM had crossed the Mediterranean Sea in boats to reach Europe, the other 16.7% (*n* = 8/48) indicated to have fled through overland routes only.

### Clinical diagnoses

In 129 out of the 154 URM (83.7%) a clinical diagnosis statement was explicitly given by the examining physicians, Table 1 shows the diagnoses in their order of frequency.

**Table 1:** Prevalences of main clinical diagnoses

Diagnosis	*N*	%
Scabies	*n* = 43/154	27.9%
suspected PTSD	*n* = 7/28	25.0%
TB	*n* = 9/154	5.8%
Pediculosis	*n* = 8/154	5.2%
Bronchitis	*n* = 3/154	1.9%
Other	*n* = 34/154	22.1%
No clinical findings	*n* = 58 /154	37.6%

In some participants more than one diagnosis was documented. Therefore the percentages add up to more than 100%. The suspicion of PTSD was raised in locations A and B only, however, since a structured assessment was only done in location A, only suspected cases from this location are listed. The category “Other” comprises (in alphabetical order): alopecia, asthma, cardiac murmur, conjunctivitis, ectoparasites (non-pediculosis, non-scabies), eczema, enterobiasis (clinical), eye trauma, gastroenteritis, hernia, hypertension, hypotension, jaundice, leishmaniasis, lymphadenitis, malaria, orchitis, scoliosis, skin ulceration, soft tissue abscess, thrombocytopenia.

Not explicitly mentioned in Table 1 are 2 cases of Female Genital Mutilation (FGM) that were found among the 9 female participants. They were 14 and 18 years of age and both coming from Somalia.

Altogether 49 of the 154 URM (31.8%) had a dermatological finding, in 27.9% (*n* = 43/154) individuals the clinical diagnosis of scabies was recorded. In 9.1% (14/154) the individuals reported itchiness. All 4 referrals that were made to a dermatologist were due to scabies. Other findings included 2 cases out of 154 (1.3%) suspicious of cutaneous Leishmaniasis, one atopical eczema (0.6%) and one leg ulceration (0.6%).

Almost three quarters (71.4%; *n* = 30/42) of the children with a recorded dental status, were classified as needing an intervention through a dentist, with 3 of them being directly referred to a dentist.

Within the cohort, 58 URM (37.6%; *n* = 58/154) had no clinical findings. However, after considering all laboratory results, the TB diagnostics and the referrals to specialists only 12.3% (*n* = 19/154) had neither a recorded diagnosis, nor a pathological technical finding nor a recorded suspicious symptom.

### Infectious diseases

#### Hepatitis a

In location A in 57.1% (*n* = 16/28) the anti-HAV IgG only was determined, and in 75% of these (*n* = 12/16) the result was positive. In location B only in 2 URM the anti-HAV IgG was determined, in both cases with a negative result. In location C both the anti-HAV IgM and the anti-HAV IgG were measured. All anti-HAV IgM were negative and all anti-HAV IgG were positive in the whole sub-cohort (*n* = 65/65). Across all locations, 92.8% (*n* = 77/83) of the URM showed immunity against hepatitis A.

#### Hepatitis B

In a total of 73.4% (*n* = 113/154) of the participants all three variables HBsAG, anti-HBs and anti-HBc were available. Among these, 8.0% (*n* = 9/113) were HBsAG positive and therefore considered to be chronic carriers of hepatitis B virus, although a recent infection and hence an acute HBV infection cannot be ruled out. In 92.0% (*n* = 104/113) HBsAg was negative. Only anti-HBs positive were 4.4% (*n* = 5/113), and 30.1% (*n* = 34/113) were anti-HBc positive regardless of anti-HBs status, and therefore considered to have been previously exposed to hepatitis B virus in the sense of a resolved infection. In 57.5% (*n* = 65/113) of the tested participants all three variables were negative and they were therefore considered naive with regard to exposure to hepatitis B virus. The following pie chart shows the HBV exposure status.

**Fig. 3:**
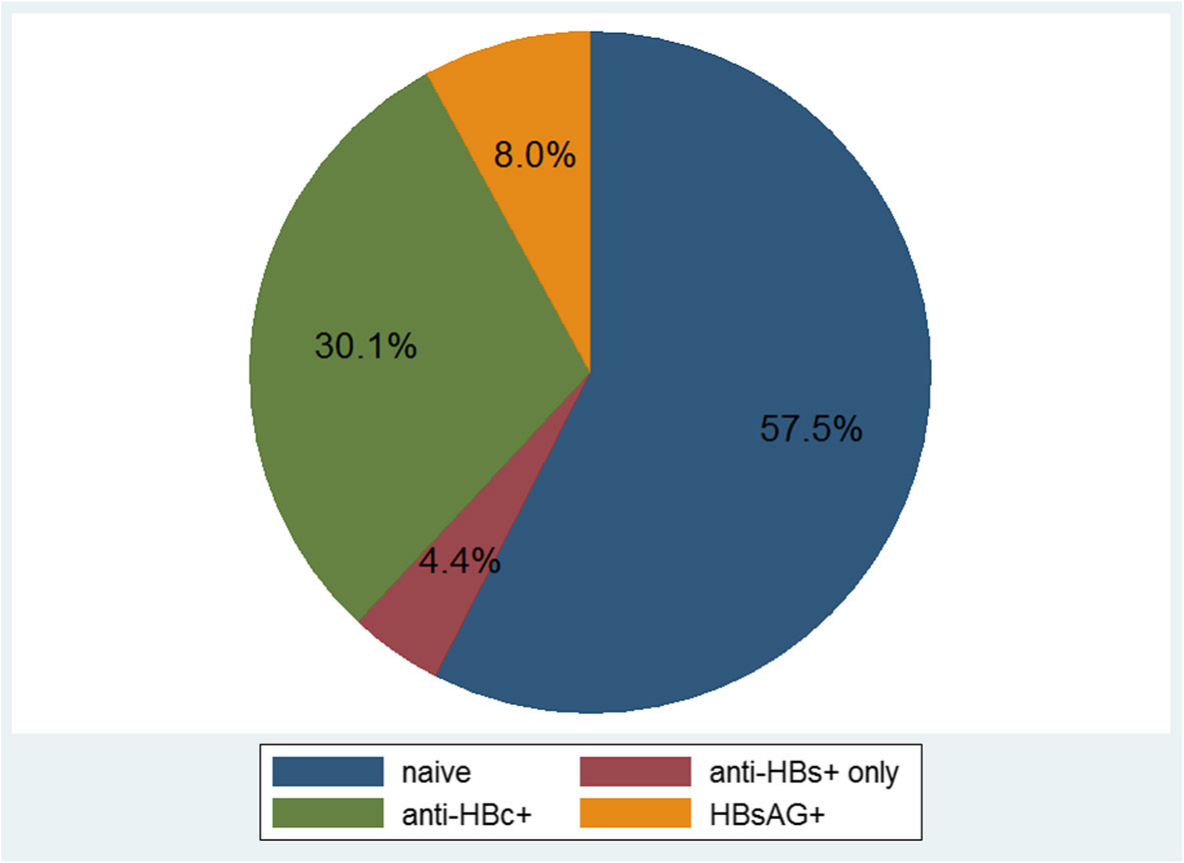
HBV exposure status. The pie chart represents all participants in whom the complete HBV serology was determined

In location C exact anti-HBs levels were determined. In two URM classified as anti-HBs positive only, the anti-HBs levels were 23 and 119 IU/l, respectively (Fig. 3).

#### Hepatitis C

In location C 100% (n = 65/65) of the UMR were tested for hepatitis C antibodies. One male (1.5; *n* = 1/65), a 16 year old URM had a positive result.

#### HIV/syphilis

In 81.8% (*n* = 126/154) of the children the HIV status was determined and a syphilis screening through a TPHA test conducted. In all individuals the result for HIV was negative, while in 2.4% of the URM (*n* = 3/126) the TPHA test revealed a positive result; all 3 were male in the age range between 16 and 17 years.

#### Screening for pulmonary tuberculosis

The screening for tuberculosis encompassed multiple variables, including laboratory results, chest X-Ray results, clinical symptoms and the physicians’ judgements. The availability of these different aspects was different in each location.

In location A previous contacts to known Tb cases was documented in 82.1% (*n* = 23/28) of the individuals, with 25% (*n* = 7/28) stating to have had respective contacts. In addition, Quantiferon Gold Test was executed in 78.6% (*n* = 22/28) individuals, with one result (3.6%) returning positive in a URM who has not indicated prior Tb contact. No suspicion of tuberculosis appeared in the list of diagnoses here, no chest X-Rays were documented, and cough as an indicating symptom was not routinely documented in this location at all.

In location B the examining physician has been a specialist for pulmonology. Here both X-Ray and Quantiferon testing were conducted. In addition cough was documented. A total of 9.8% (*n* = 6/61) of the URM were classified as suspect cases for Tb. Besides these suspect cases there were 8.2% (*n* = 5/61) of the URM with a documented positive Quantiferon test, which could be assumed to be latent infections.

In location C there were neither Quantiferon test nor chest X-Ray conducted. However in 4.6% (*n* = 3/65) of the URM the suspicion of a case of tuberculosis was expressed and a referral to a specialist for pulmonology ordered.

In 5.8% (*n* = 9/154) of the entire cohort suspected cases of tuberculosis were identified and 3.9% (*n* = 6/154) were supposedly latent infections.

#### Stool analysis

Routine stool diagnostics was conducted in 55.2% (*n* = 85/154) of the URM, while in 7.1% (*n* = 11/154) a parasitological finding was obtained. In 5.2% (*n* = 8/154) of the participants Gardia lamblia was identified, in 1.3% (*n* = 2/154) of the participants Schistosoma mansoni and in 0.6% (*n* = 1/154) of the participant Strongyloides stercoralis. In a total of 40.3% (*n* = 62/154) of the children an antigen test on Helicobacter pylori was performed in the stool sample, returning a positive result in 25.9% (*n* = 40/154).

#### Haematological results

In 72.7% (*n* = 112/154) of the URM haemoglobin (Hb) was measured. In location A and B no anaemia was found, based on their threshold definition of 11 g/dl. In location C, 40.6% (*n* = 26/64) were classified as anaemic. However, this classification is based on higher threshold values as compared to locations A and B (see section “methods”). If the same threshold level as for locations A and B was to be applied, only 3 individuals would have been declared as anaemic. No pathological findings were recorded for the mean corpuscular volume (MCV).

An increased eosinophil count was measured in 17.4% (*n* = 8/46) of the participants where differential blood count was performed. None of the individuals with intestinal parasites showed an eosinophilia.

The level of the leukocyte count was measured in 98.5% (*n* = 64/65) of the URM in location C only. The mean count was 6.61 × 10^3^/μl (SD = 2.02). Only 3.1% (*n* = 2/65) of the URM had leukocyte counts above 10.0 × 10^3^/μl. Their diagnoses were scabies and an ulceration of the leg.

Thrombocyte counts as well were only available in location C. Here, in 98.5% (*n* = 64/65) of the URM the mean count was 288 × 10^3^/μl (SD = 76; range 113 to 476).

## Discussion

For the extraordinary influx of refugees in 2015 in Bavaria there are no scientific data available [3]. Investigative journalism has estimated the arrival of more than 2300 URM in one district of Bavaria where two of the locations are settled [19]. Currently it is expected that an increased number of URM will continue to arrive to Germany. Through the three datasets presented it becomes very obvious that at the moment there is no standardized concept for clinicians regarding the first medical examination of URM, although several initiatives to establish standardized procedures have been developed in Germany, such as the Bremer Untersuchungsheft or the Jugendvorsorgeuntersuchung für Unbegleitete Minderjährige Flüchtlinge (J-umF, screening examination for URM) [20, 21]. Personal communications with the involved physicians revealed that the spectrum of investigations to be conducted in the primary examination of URM had to be discussed on an individual basis with the representatives of the Youth Welfare Office. Moreover, these regulations also changed over time, leading to changes in the algorithms for laboratory diagnostics within the same location. In these examinations data about the growth and pubertal development of the URM were not considered. The reasons behind were on one side time and capacity limitations, but also the lack of possibility for further follow-up of the URM, because of re-distribution of the URM to other German states.

Every clinician had to develop his own examination form, as templates were not available. The extent of clinical examinations itself was seemingly carried out depending on individual experiences by each examining physician. These findings are calling for a common documentation system and standardized guidelines for all examining physicians in refugee settings. Furthermore it has to be mentioned that all examinations took place in addition to the daily work of the well frequented practices of the individual physicians. The physicians themselves applied a very basic paper-based documentation of the results, but the URM did not receive any documentation to be handed on to health care providers at their final point of settlement.

About one third (37.6%; *n* = 58/154) of the URM had no clinical findings. However, this subgroup of individuals diminished in numbers when all laboratory results, the TB diagnostics and referrals were included. Therefore a high overall morbidity of 87.7% with at least one clinical diagnosis or suspicious laboratory result was seen in this cohort, which is relatively high compared to data from Berlin where 56.6% had no pathological finding [22]. A study on microbiological screening in Hamburg, Germany found pathogenic and non-pathogenic microorganisms in apparently healthy URM [23], while in Western Australia over 90% of the participants in a voluntary health assessment were referred to special, multidisciplinary paediatric health clinics [24]. The majority of the resulting health care needs in this refugee population are known to general practitioners in Germany. These findings correspond to studies carried out in a collective refugee housing in Munich and to a reception centre for refugees in Italy [25, 26]. Nevertheless, also uncommon findings have to be expected, like e.g. in this cohort two cases of clinically diagnosed Leishmaniasis. These diseases have to be correctly diagnosed or in the case of uncertainty referred to specialists, in order to provide adequate treatment in a timely manner. The haematological findings such as platelet or white blood cell counts are seen as formative for future health professionals involved in service provision to URM, and indicative for certain disorders, like visceral Leishmaniasis or immunosuppressive disorders. Recently, publications are appearing that are focusing on particularities of clinical findings in refugee populations, such as haematological parameters [27].

Compared to previous studies in Germany on the hepatitis A immunity of refugees [28] this cohort had even a higher prevalence (92.8%) of HAV immunity. In the cohort of Jablonka et al. the immunity in the age group below 18 was 81.1%. Based on these figures, general vaccination campaigns for hepatitis A seem not to be a priority in these population groups. Still hepatitis A immunity needs to be monitored in different settings in order to verify that the paediatric populations are sufficiently protected.

The status of immunity for hepatitis B was indicated through different serology constellations. In location C the exact anti-HBs levels were determined. This data allows the interpretation that only 3.2% (*n* = 5/154) of the children seemed to have been vaccinated in the past. Only in one (0.6%; *n* = 1/154) of the URM the level was high enough to assume long-term protection against hepatitis B [29]. The low level of long-term protection and the large proportion of naive individuals make a general immunization of newly arriving URM against HBV reasonable. This is indicated especially in crowded housing conditions where a higher incidence of horizontal transmissions has to be expected. However, these conclusions have to be taken with some degree of caution due to general limitations of seroprevalence studies, which are for example unable to detect occult HBV infections.

A total of 64.5% (*n* = 40/62) of the URM tested for Helicobacter pylori were positive. A previously conducted study described that mortality through e.g. stomach cancer is increased in migrant populations. Spallek et al. explained this risk through a widespread infection with Helicobacter pylori in children in developing countries [30]. This cohort as well presented a high prevalence of this infectious agent. This may be taken as one example for the importance of adequate continued health care for URM after a first stage of arrival in the host country with regard to potentially arising future health problems.

Two out of nine of the female URM (22.2%) in the cohort indicated to have undergone FGM. Especially with regard to the known countries of origin of all female URM of the cohort (Somalia and Eritrea), this low prevalence is questionable. In a report from the Population Reference Bureau the prevalence of undergone FGM for females of the age group between 15 and 19 years was indicated for Somalia at 96.7% and for Eritrea at 78.3% [31]. The medical condition was detected through taking of medical history only, no gynaecological examination was done. The dependence on self-reporting could be one of the explanations for the rather low prevalence of FGM in the data. Due to the sensitivity of the topic it can only be speculated that the female URM may have been reluctant to disclose, particularly since the attending clinicians were all male. An additional, general factor that may have inhibited disclosure of medically relevant information from the side of the URM may have been the rapidity of the patient-health care staff contact, and the absence or deficient qualification of interpreters, as has been pointed out in other publications, such as a recent review by Van Os et al. [32].

The risk to develop PTSD is depending on the kind of trauma. About half of the victims of war, torture or flights are expected to develop PTSD, compared to about 10% of victims of traffic accidents, according to a guideline on PTSD from 2011 [33]. In this study one quarter (25%; *n* = 7/28) of the URM from location A have suffered from a suspected post-traumatic stress disorder. Other studies analysed the prevalence of PTSD in children of refugees in Germany and showed that 19% had the full clinical picture of PTSD [34]. Previous studies showed a remarkable level of stressful life events in URM as compared to accompanied refugee minors, e.g. in the experience of physical maltreatment in 63.3% of cases as compared to 23.2%, respectively [35]. URM have to travel alone or get separated from their caregivers during the flight and are in consequence particularly vulnerable through exposure to dangerous situations and abuse [36, 37]. Previous studies mentioned that URM are more likely to show self-harm or suicidal behaviour than no-URM [38].

It has to be mentioned as a shortcoming of this study that PTSD itself was not diagnosed in this sub-cohort by a physician particularly trained in the detection of PTSD but by applying clinical judgement only, based on three key symptoms. The diagnosis should be more interpreted as an assessment of distress by addressing indicators comparable to a quick PTSD assessment. Nevertheless, these core symptoms are matching with the contents of commonly used questionnaires, such as the child PTSD symptom scale of the American Academy of Child and Adolescent Psychiatry [39]. The focus was more a rapid assessment of the problem magnitude in the target population than the individual diagnosis, similar to a study from Sweden, where the usefulness of a self-reporting questionnaire was analysed [40]. A study from Western Australia recommended the development of longitudinal studies to learn about the connection between previous suffering and the mental health outcome of refugee children [41]. It has to be kept in mind that the contact time between the physician and the examined URM was very low, and options for follow-up were practically non-existent, also due to a rapid allocation of refugees to different parts of Germany.

An additional weakness of the study is the data pooling of all three sites for the data analysis, instead of the analysis location by location. It is very unlikely that the distribution of the URM to certain locations for their medical entry examination was geared by a substantial bias. It was assumed that the URM that entered Bavaria in the investigated time period represent a collective target population with comparable backgrounds.

Certainly the disparities between the different assessment tools that were applied by the involved study sites and physicians may be summoned as a weakness of this study. However, the authors see this aspect rather as a finding of the study, and hope to contribute hereby to the future development of standardized tools to be employed in similar situations in the German health system when working with unaccompanied refugee minors as patients.

## Conclusion

This study shows the spectrum and extent of immediate health care needs of URM upon their arrival to Germany. The majority of the health conditions are common illnesses known to German physicians. At the same time the findings have to be interpreted in the light of the peak influx of refugees into the German state of Bavaria towards the end of 2015. Two key findings are the lacking of standardized algorithms and documentation for the medical coverage of URM in Germany, and considerable potential for primary and secondary prevention of illnesses through evidence based health interventions, such as vaccination against hepatitis B or psychosocial support in populations prone to suffering from PTSD.

## Abbreviations

PTSD: Post Traumatic Stress Disorder
URM: Unaccompanied Refugee Minor
